# Historical influence on the practice of chiropractic radiology: part II - thematic analysis on the opinions of diplomates of the American Chiropractic College of Radiology about the future

**DOI:** 10.1186/s12998-017-0145-z

**Published:** 2017-05-08

**Authors:** Kenneth J. Young

**Affiliations:** School of Arts, School of Health Professions, Murdoch University, Perth, WA 6150 Australia

## Abstract

**Background:**

Over the past 20 years, various authors have addressed the question of the future of chiropractic. Most were positive about the future, with some advocating evidence-based practice and integration with mainstream healthcare, some advocating continued separation with an emphasis on subluxation-based care or the traditional/historical paradigm of chiropractic, and some calling for tolerance and unity. No papers were found specifically inquiring about the future of chiropractic radiology.

**Methods:**

The study population consisted of all current members of the American Chiropractic College of Radiology (ACCR), estimated at 190 people, known as chiropractic radiologists or Diplomates of the American Chiropractic Board of Radiology (DACBRs). An internet-based, anonymous survey using SurveyMonkey was implemented, supplemented by hard copies distributed at a conference. The main point of interest for this paper is the final item of the overall questionnaire. This item inquired about the future of chiropractic radiology. Thematic analysis was used on the responses, coded in both constructionist and inductive ways to extract both a general outlook and more specific themes. The inductive themes were also assigned secondarily to a SWOT (strengths, weaknesses, opportunities, and threats) analysis.

**Results:**

The overall response rate to the survey was 38% (73/190); within the group of respondents, 71 of 73 (98%) answered the item that is the subject of this paper. Opinions on the outlook for chiropractic radiology in the future were more negative than positive, with 14 respondents giving a positive outlook, 26 negative, and 14 non-committal. 28 respondents advocated integration with the wider healthcare community, 11 recommended emphasising separateness or a focus on working within chiropractic, and 15 did not express an opinion on this issue. Ten strengths were noted, 11 weaknesses, 57 opportunities, and 30 threats.

**Conclusions:**

The increasing necessity of demonstrating evidence for diagnostic and therapeutic procedures in healthcare makes it likely that chiropractic radiologists and the wider chiropractic profession will need to take a more active position on evidence-based practice. Re-evaluation of guidelines and legislation as well as enforcement policies and practices will be necessary. The consequences of failing to do so may include increased marginalisation and reduced viability as a profession.

**Electronic supplementary material:**

The online version of this article (doi:10.1186/s12998-017-0145-z) contains supplementary material, which is available to authorized users.

## Background

This study originated as part of a survey exploring the influence of historical chiropractic radiography/radiology paradigms on the current practice of Diplomates of the American Chiropractic Board of Radiology (DACBRs or chiropractic radiologists). The final item in the survey asked the respondents for their opinions on the future. Commentaries on the future of chiropractic can be found in the scholarly literature and professional magazines over the past 20 years [1–37]. Some of them advocated that the way forward lay in a non-evidence-based mode, that is, vitalism [6, 22, 28, 31], subluxation-based practice [4, 22, 28, 31] or chiropractic as alternative/primary care [3, 31]. Others emphasised evidence-based chiropractic with integration into mainstream healthcare as the path for success [1, 7, 9, 10, 12, 14, 16–18, 20, 21, 23, 24, 32, 33]. Some expressed a need to unify the profession to ensure its survival and growth [2, 6, 8, 11, 25, 27, 36]. These ‘unitarians’ promoted the idea that chiropractors should be tolerant and inclusive of the wide variety of practice paradigms that existed, and that divisiveness within the profession was destructive. Simpson [17] took the alternative view, advocating that chiropractic split into two professions, with both the vitalistic and evidence-based sides clearly delineating their terms. Authors voiced sentiments of pride in various achievements, with the victory of the anti-trust ‘Wilk’ trial [11, 17, 26, 27, 30], increasing mainstream integration (e.g., with multidisciplinary healthcare teams and insurance companies) [3, 5, 7, 14, 15, 21, 23, 29, 30, 32, 33], increasing presence in universities [1, 10, 16, 20, 29, 33], or the slow but definite progress in research [1, 7, 9, 10, 16, 19, 26, 27, 29, 33, 35] as frequent topics.

Some were broad-based in their consideration while others focused on one area such as education, clinical practice, or research. Although all admitted that challenges existed, the vast majority were positive about the future. One exception to this was Mootz in 2007 [37], who predicted possible extinction and replacement of chiropractors by Doctors of Physical Therapy in the United States of America (USA). He suggested a move toward evidence-based practice, research priorities oriented toward best practice, constructive engagement with mainstream healthcare and ethical business models.

All the articles listed above [1–37] offered bits of advice for the future, but Triano et al. [33] developed a detailed plan. In 2006 Triano and others convened the Chiropractic Strategic Planning Conference, calling on experienced leaders in the profession under a steering committee of three people chosen for their mainstream academic credentials (i.e., JD, PhD, and MSc qualifications in addition to chiropractic degrees). They made specific recommendations within five domains: education, research, regulation, workplace, and leadership. The resulting document emphasised the importance of reflective learning in the profession. This was correlated with the fact that the privilege of self-regulation was contingent on maintenance of the fiduciary contract that elevates patients’ interests above those of practitioners’. It was noted that self-regulatory privileges could be withdrawn by a society from a profession that failed to abide by this principle. To minimise this possibility, the authors recommended high standards of ethics in clinical and business practices, engagement with the political process, increasing collaboration with the wider healthcare community, and fostering cultural authority.

One of the major efforts in forecasting the fate of the profession was commissioned in 2013 for the Institute for Alternative Futures (IAF) by the National Chiropractic Mutual Insurance Company (NCMIC) under the guidance of its president, Louis Sportelli. This was the third document of its type produced by IAF since 1998. The report conceived four possible futures for chiropractic, which they called scenarios. Scenario 1 was called ‘Marginal Gains, Marginalised Field’. It essentially involved attempting to support the subluxation-based model with research but because of the narrow focus the outcome was poor. This scenario predicted the closure of some chiropractic teaching institutions, low starting income for chiropractors, limited career prospects, high student debt, and limited growth of the profession. Scenario 2 was called ‘Hard Times and Civil War’. It was predicated on a second recession in 2015 and a continuance of the schism in chiropractic between evidence-based and subluxation-based chiropractors. It suggested a worse outcome than Scenario 1. The IAF called Scenario 3 ‘Integration and Spine Health Leadership’. In it chiropractors embraced the role of spinal health expert and joined with mainstream medicine. The prediction was for sustainable or even thriving solo and group practices as well as expanded chiropractic legislation. Scenario 4, ‘Vitalism and Value’, involved a growing popularity for vitalism. It predicted that chiropractic could fit into this movement and focus on wellbeing and health promotion. But lack of supporting research and market financial structures limit growth of the profession [38].

A study by Gliedt, et al. [39] revealed that chiropractic students in North America wanted the future to hold mainstream acceptance for the profession (87%) as well as maintenance of traditional chiropractic principles, including emphasis on ‘correction’ of vertebral subluxation (61%). The authors recognised that this must either represent cognitive dissonance or the desire to engage in scientific research on the possible effects of subluxation. It seems as if many chiropractic students would like fringe views to be accepted by the mainstream. However it is also possible that there was little overlap between the two groups. It may be that a larger proportion of chiropractic students held one but not both views. This study also suffered from a 17% response rate, and so it may not be generalizable to the general population of chiropractic students.

Of all the studies encountered [1–34] only one article specifically addressed the future of chiropractic radiologists or the future of diagnostic imaging for the larger profession of chiropractic. In that article, Yochum [9] addressed only technical advances in equipment. It was thought that a survey of those people with the most knowledge of diagnostic imaging in the profession, chiropractic radiologists, might be able to provide unique insights. Specifically, information was sought as to the effects of the historical paradigm of radiography in chiropractic, that is, subluxation detection, on the current practice of radiography in the profession, the practice of chiropractic radiologists, and the potential future of imaging in chiropractic. It was hoped that data could be elicited relating to adherence to x-ray safety protocols and use of radiographic guidelines by chiropractors as well as other nuances of practice not captured in quantitative studies of practice methods like the NBCE practice analysis reports [40]. A survey was taken of DACBRs and the results of most of the items are related in Part I of this linked pair of papers. During the data analysis of the questionnaire it became apparent that respondents had strongly engaged with the final item, which asked them for their opinions on the future of radiology in chiropractic. The respondents provided thoughtful, detailed written answers about their opinions on the subject. It was determined that in order to give these responses proper consideration, they should be presented on their own, using a qualitative research method known as thematic analysis.

Thematic analysis is useful for questions related to experiences, views and perceptions. It involves searching a data set for elements of relevance or ‘themes’. Braun and Clarke [41] defined themes this way: ‘A theme captures something important about the data in relation to the research question and represents some level of patterned response or meaning within the data set.’ Themes are then categorised or ‘coded’ into groups that can help identify patterns of meaning to help answer the research question. The process involves a thorough familiarisation with the data, coding, theme development, and revision. Table 1 provides the thematic coding.

**Table 1:** Ways of coding themes for analysis [41]

Inductive	Coding and theme development are directed by the content of the data
Deductive	Coding and theme development are directed by existing concepts or ideas
Semantic	Coding and theme development reflect the explicit content of the data
Latent	Coding and theme development report concepts and assumptions underpinning the data
Realist	Focuses on reporting an assumed reality evident in the data
Constructionist	Focuses on looking at how a certain reality is created by the data

## Methods

Approval was obtained from the Murdoch University Human Research Ethics Committee (approval number 2015/142). The study population consisted of all current members of the American Chiropractic College of Radiology (ACCR), estimated at 190 people. All active DACBRs are ACCR members. An internet-based, anonymous survey using SurveyMonkey was implemented, based on a modified Dillman method [42]. First, a herald notice was sent one week before the questionnaire was opened on the internet in order to inform the recipients of the purpose of the study, stimulate their interest and ask for their cooperation. Then an online survey link was sent directing the recipient to the Information Letter, Informed Consent, and the questionnaire. Two reminders followed at bi-weekly intervals. Recipients who had already completed the questionnaire were asked to ignore the reminders. The Secretary of the ACCR sent all the emails to the ACCR members. By coincidence, the author was scheduled to attend the annual conference of the ACCR during the time of data collection. Hard copies of the survey were printed and one announcement was made to the conference attendees during a break between lectures, notifying them of the availability of the survey, if they had not previously filled out an online version. In order to maintain the anonymity of respondents, questionnaires were left at the conference registration desk, and the receptionists agreed to distribute copies and collect completed ones.

The last item of the survey (Item 34) asked of DACBRs the following: What do you think the future holds for the practice of chiropractic radiology? Responses from the online version were cut and pasted from the SurveyMonkey results form into a Microsoft Word document. Results from the hard copies were transcribed into the same document. All responses were kept separate under respondent numbers. Responses were minimally edited for spelling and grammar without changing the meaning. All responses were read several times until familiarity was reached. A Microsoft Excel spreadsheet was created for coding the data with the respondent number down the left column and themes as headers for the remaining columns. It was determined that all responses could be coded as constructionist themes in two ways. First, they were coded for basic outlook: positive, negative, or non-committal. Second, they were coded by the respondents’ opinion on the best path forward: Mainstream, chiropractic/separate, or unknown. ‘Mainstream’ meant that respondents had expressed the idea that chiropractic should embrace mainstream healthcare or evidence-based practice. ‘Chiropractic/separate’ meant respondents favoured traditional chiropractic ideas or forging a path independent of mainstream healthcare. Further, all primary themes, those that emerged as written by respondents, were also coded on the spreadsheet in an inductive way. After they were entered as they had occurred in order of respondent it was realised that they could fit a SWOT (strengths, weaknesses, opportunities, and threats) analysis, and so they were re-ordered under these secondary themes. A number ‘1’ was entered into the box under a theme if it appeared in a response. This way, the number of times a theme appeared could be automatically totalled along the bottom row. Several statements were ambiguous, but it was decided to list any one sentence only under one inductive code, rather than having statements appear under two codes, thus inflating the apparent number of themes elicited.

## Results

The overall response rate to the survey was 38% (73/190); within the group of respondents, 71 of 73 (98%) answered the item that is the subject of this paper. Additional file 1 contains all the responses. Table 2 displays the constructionist theme ‘outlook’. Table 3 displays the constructionist theme ‘path forward’. They both include the number of times each theme appeared and representative examples of each type. Overall, the outlook was more negative than positive, and advocacy of mainstreaming appeared more than support for separateness.

**Table 2:** Outlook

Outlook	Positive	Negative	Non-committal
Quantity	14	26	14
Examples	- I believe there is a place for chiropractic radiology… -Future’s so bright, I gotta wear shades.	- I feel the future of chiropractic radiology is precarious at best… - I think it is grim. There are many radiologist now that are specializing in MSK imaging and understand biomechanics.	- The practice will largely be dependent on financial structures, coverage and reimbursement. - No idea about the future, or how (at least in the US) how healthcare changes will affect it.

**Table 3:** Path forward

Path forward	Mainstream	Chiropractic/separate	Unknown
Quantity	28	11	15
Examples	- It behooves chiropractic radiologists to affiliate with these imaging centers to overread the medical reports. - Chiropractic radiologists didn’t evolve for subluxation analysis, but as diagnostic specialists.	- Many of my colleagues want to be radiologists not chiropractic radiologists. - Spinal images, if available, should be routinely evaluated for biomechanical findings as these may affect patient prognosis which is a part of the management.	- In what country? The answer differs depending on the jurisdiction. - There will only be handful of chiropractic radiologists working in a full time practice solely dedicated to radiological consultation.

Table 4 displays the inductive themes, the number of times they appeared and representative examples of each type. Ten strengths were noted, 11 weaknesses, 57 opportunities, and 30 threats. Three responses were considered not applicable to any of the inductive themes. The strengths were inherent to DACBRs for the most part, such as their perceived strength as teachers, and the respect sometimes offered by the medical community because of their abilities. Whereas, the weaknesses identified were not with DACBRs but rather characteristics of chiropractors outside this group, predominantly the undervaluation of DACBRs by clinically practicing chiropractors. Opportunities generally focused on involved integration with the wider healthcare community and technology. Threats were also identified as being outside chiropractic: from the medical community, reduced plain radiography due to advanced imaging techniques, and reduced insurance reimbursement.

**Table 4:** Inductive themes with representative examples of responses

Category of theme	2^nd^ degree theme	1^st^ degree theme	Qty	Examples
Inductive themes	Strengths	Collegial respect	2	- 20% of my full time practice is requests from health practitioners other than chiropractors. So they respect our competency and education. - I am treated as an equal from the medical radiologists.
		More detail in reports	1	- More detailed report with findings sometimes overlooked by our MD radiologists brethren.
		Improved chiropractic education	2	- I believe that the chiropractic profession in general improves over time as education has improved. - The demand for chiropractic radiologists will grow as the chiropractic clinicians skill set grows with the management of more challenging clinical problems.
		Perseverance	1	- We make our opportunities by working our rear-end off, ethically and without compromise…and with a whole lot of patience & humility.
		Teaching	4	- Most will likely need a full time work whether in a clinical or academic setting. - Training radiologists to teach at the institutions.
	Weaknesses	Image quality	2	- It seems most chiro offices that have x-ray suites produce garbage film quality. - [Institutions] should concentrate on [teaching] good film quality.
		Undervaluation of DACBRs	8	- The value of [DACBRs] seems to be ignored by 90% of practicing Chiropractors. - I worry that we are not valued enough by the chiropractic profession.
		Vitalism	1	- Those practitioners who operate in a more vitalistic model are not concerned with pathology and do not believe expert interpretation worthwhile.
	Opportunities	Accreditation	6	- Some residencies will be phased out due to the cost of meeting stringent external accreditation expectations. - We will have to get some form of external accreditation for our training and certification process or ACR will push us out of the sandbox.
		Advanced imaging	9	- More MRI: the chiropractic radiologist is going to have to be credentialed mainstream for reimbursement purposes. Chiropractic radiology training/certification for MRI reading lacks structure, lacks a credentialling process, etc. - We will need to become more capable of accessing advanced imaging facility referrals.
		Teleradiology	4	- Teleradiology issues will need to be ironed out for our profession as private practitioners accessing digital equipment will make image transference easier and more real time. - Chiropractic “radiology” will survive, but will move towards 100% digital environments and probably online/distant/international interpretation services.
		DACBRs must focus on chiropractors	10	- New DACBRs should be taught to have more of a service attitude toward treating chiropractors. - Contemplation of CMT [chiropractic manipulative therapy] can be an indication for radiographs. - I think we are remiss if we expect chiropractors to apply medical guidelines for imaging indications. - It would appear to me that there are many academic leaders who are almost totally devaluing and discrediting the need for chiropractors to order or take imaging studies. These efforts must be vigorously opposed since it is this privilege that mostly sets our system of patient care apart from all others (pists and paths) [sic].
		Integration	12	- Important for us to partner with outpatient imaging centers, or even hospitals. - Hopefully towards the trend of being further and more widely incorporated into allopathic medical imaging centers.
		Evidence-based practice	7	- I would like to see [fluoroscopy] gone - it does not offer medical benefit. - If the profession as a whole can move towards working in an evidence-based manner, then there may be a future for chiropractic radiologists. - We have to be aware of and contribute to the current literature with respect to the significance of imaging findings.
		Future depends on future of chiropractic	7	- As the profession goes, so do we - As a radiologist who reads films for chiropractic practices… continuing growth. - Specialized fields (like chiropractic radiologists) depend on the health of the referring body of chiropractors.
		Incorporate interventional procedures	1	- Imaging groups… are less likely to hire chiropractic radiologists who can’t perform basic interventional procedures.
		Political representation needed for DACBRs	1	- We need a strong independent DACBR professional organization to represent us a distinct speciality.
	Threats	Extinction	5	- It will eventually die out, not in my or your lifetime. - I would not be surprised to see the field completely disappear in 20 to 30 years.
		Medical prejudice	3	- Continual efforts by organized medicine to exclude DACBRs from film reading opportunities will also make it more difficult to remain viable. - MD radiologists are pressured by their colleagues to not allow DC radiologists to practice with them.
		Less radiography	8	- As fewer chiropractors take x-rays, those patients who DO need x-rays will be sent to imaging centers who have their own medical radiologists. - As evidence based medicine and collaborative care continues to gain popularity, how long will it be before chiros in North America also lose their right to use (abuse) ionizing radiation as a diagnostic tool?
		Reduced reimbursement	14	- Most chiropractors seem less inclined to pay for radiological interpretation services, and those who are inclined, many times cannot afford the expense in a managed care model of private practice. - Majority of my reads are cash basis because insurance reimbursements are so poor.
	Not applicable	Answer too complex to give on survey	3	- Too long to explain. - This question is too broad. Respectfully, I don’t have time to tell you everything I think about this topic.

## Discussion

Ninety-eight percent of respondents to the main questionnaire (Part I) answered the last item, which is the subject of this paper. Considering that there was quite a bit of random item skipping throughout the rest of the questionnaire, this would seem to indicate strong interest in the subject of the future of chiropractic radiology. The outlook was predominantly negative, but respondents recommended collaboration with mainstream healthcare as the best path forward; this fits with the ever-increasing emphasis on evidence-based healthcare in general. Strong threats were identified, but also many opportunities. These two findings, in conjunction with a predominantly negative outlook could indicate the opinion that the profession may fail to fulfil the potential that it is perceived to have, that it may be held back somehow. Although there was nearly the same number of strengths as weaknesses, the weaknesses seemed more significant in content. In particular, the perception that DACBRs were undervalued by the rest of the profession was prominent. This may be due to the fact that there are very few DACBRs, and that there are some chiropractors that do not use radiography primarily for pathological diagnosis, but rather for the historical paradigm of subluxation detection. Each set of themes will be considered.

### Constructionist theme - Outlook

In thematic analysis, constructionist themes interpret a reality created by the data. In the current case, the responses generated both an outlook, and a path forward. The outlook was defined as a prediction of the outcome for the profession of chiropractic radiology. In other words, respondents gave their view of the future, and ways in which their role as diagnostic imaging specialists could be preserved or improved. Upon examining the data, these two constructionist themes were readily seen. In most cases a positive or negative outlook was obvious from the response, and the negative was more commonly found than it was in the articles referenced earlier in this paper. Two factors may be relevant here. The first is that writing an opinion piece for a magazine article is different from completing an anonymous survey. People might not feel free to express their real sentiments when they write under a true by-line. The other factor is that chiropractic radiologists are a distinct group among chiropractors, and hence have different forces operating on their livelihoods. First, DACBRs lack official recognition for their speciality from accrediting bodies and registration/licensing boards, although there are special interest groups in various organisations like the European Academy of Chiropractic [43], and the Royal College of Chiropractors (UK), which has a specialist faculty in imaging [44]. This creates a disadvantage in marketing themselves to chiropractors as well as in competing with medical radiologists. In addition, DACBRs cannot call themselves simply ‘radiologists’ because this may be interpreted as indicating that they hold a medical qualification, which is specifically forbidden by law in places [45–48]. There are also challenges to reimbursement. DACBRs cannot be reimbursed for radiology reporting under Medicare or Medicaid in the USA, in the National Health Service in the United Kingdom, or under Medicare in Australia, and private insurance is variable in its reimbursement around the world. Private imaging centres have little reason to employ a chiropractic radiologist when a medical radiologist can perform all the same functions, plus all types of advanced imaging interpretation, and potentially even invasive investigatory and interventional procedures such as MRI with gadolinium, CT with contrast or ultrasound guided cortisone injections into joints and bursae. Respondents to the current survey noted this with two mitigating factors. It was observed that DACBR reports tended to be more detailed than medical imaging reports and that DACBRs tended to report biomechanical factors, with the implication that this was viewed favourably by the referring chiropractors.

### Constructionist theme – Path forward

The constructionist theme of ‘path forward’ also readily emerged from the respondents’ writing. It was thought useful to categorise this constructionist ‘reality’ created by the responses, in order to gauge an overall impression of the general direction DACBRs would like to take for the future. This theme was defined as the actions necessary to create a viable future for DACBRs. Although a negative outlook was common, respondents offered potential solutions to the problems they identified. Those that had a positive outlook also often offered ways to improve the outlook for chiropractic radiologists. In examining these solutions, the divergent ideas of either integrating with mainstream healthcare or emphasising chiropractic’s separateness divided the responses, although some remained neutral. The largest group of respondents indicated that chiropractic should integrate with mainstream healthcare. They raised ideas like incorporating interventional procedures, gaining accreditation from medical radiology associations, and working in imaging centres. One also specifically addressed the historical chiropractic radiography paradigm: ‘Chiropractic radiologists didn’t evolve for subluxation analysis, but as diagnostic specialists’. An intermediate number of respondents did not state a way forward, and the smallest number of responses on this theme advocated a more distinct path, emphasising chiropractic-specific functions for radiography. These respondents focused on the DACBRs’ knowledge of chiropractic practice and stated that this gave them an advantage over medical radiologists in interpreting biomechanical findings. This has been noted by other authors as well [49, 50]. None elaborated on how this perceived advantage was beneficial to patients, though. Two respondents mentioned that DACBRs should have more of a ‘service attitude’ towards chiropractors. One specifically denigrated the idea that DACBRs should aspire to all the functions of their medical counterparts or that DACBRs should work in conjunction with them, stating, ‘Some of the most notable accomplishments in recent years have come through upper cervical chiropractic [no references offered]… As the profession goes, so do we. As a group of imaging professionals, we do not have the numbers or the resources to directly compete with medical radiology’. These statements evoke the possible interpretation that DACBRs should be using radiographic chiropractic subluxation detection systems or employing non-mainstream biomechanical interpretations that may be lacking in evidence. Alternatively, it could have meant that because DACBRs were primarily trained in plain radiograph interpretation they should confine themselves to that, or possibly that DACBRs should focus on chiropractors as referrers instead of integrating with mainstream imaging centres. In summary, although a majority of respondents indicated integration as the best way forward, a minority who favour separateness seemed to feel strongly about that position.

### Inductive themes

As the responses were examined, and after the constructionist themes identified, it became apparent that the respondents had offered insights into what they perceived as positive and negative elements about their profession, its emergence from a vitalistic history, and how to progress. A SWOT analysis seemed appropriate to apply secondarily, in order to categorise and therefore better understand trends in the data.

### Strengths

The ten strengths in five categories identified were inherent to the DACBR community but ten did not seem like many from a total of 71 responses. Perhaps this reflected some of the overall pessimism about the future. But chiropractic radiologists took pride in their teaching abilities (*N* = 4). The idea that chiropractic education in general had improved over time (*N* = 2) also arose and was viewed positively by respondents. Respondents also cited the respect they got from medical colleagues (*N* = 2), their perseverance (*N* = 1) and the detail of their radiology reporting (*N* = 1). Anecdotally, the author has noted this last element first hand, as well as second hand in conversations with other DACBRs. It seemed somewhat surprising that this strength was not cited more frequently.

### Weaknesses

Eleven responses were coded as weaknesses, which again did not seem very many from 71 responses. Unlike the strengths, though, they were all external to chiropractic radiology, although within chiropractic. So it seemed as if DACBRs saw themselves as a strength within a profession that had some significant weaknesses, not all of which were directly related to their effects on DACBRs. The main perceived weakness (*N* = 8) was that the wider profession did not appreciate the strength of chiropractic radiologists. The other two weaknesses were the poor quality of images produced by chiropractors (*N* = 2), and the continued existence of vitalism (*N* = 1). Both have implications when viewed from outside chiropractic. Referrals of patients with poor quality images and failure to adhere to evidence-based practice, including promoting vitalism or using terminology not recognised by the mainstream healthcare community reflects badly on the entire profession of chiropractic. Since these weaknesses are not unique to DACBRs, they can be addressed in various fora such as chiropractic curricula, conferences, scholarly papers, and postgraduate seminars in order to move the profession forward.

### Opportunities

Fifty-seven opportunities were identified. With this large number of responses, it seemed as if chiropractic radiologists considered these issues in detail. Five categories together accounted for 35 of the opportunities. ‘Accreditation’ (*N* = 6), ‘integration’ (*N* = 12), ‘evidence-based practice’ (*N* = 7), ‘advanced imaging’ (*N* = 9), and ‘incorporate interventional procedures’ (*N* = 1) all involved working within the mainstream healthcare community, so this was a strong theme. The theme ‘DACBRs must focus on chiropractors’ (*N* = 10) seemed dominated by responses indicating that chiropractic radiography may be different from medical radiography, that chiropractic-specific considerations or biomechanical alterations may be legitimate justifications for the use of ionising radiation. Specifically, one respondent wrote, ‘It would appear to me that there are many academic leaders who are almost totally devaluing and discrediting the need for chiropractors to order or take imaging studies. These efforts must be vigorously opposed since it is this privilege that mostly sets our system of patient care apart from all others (pists and paths) [sic]’. This language seems to denigrate other forms of healthcare, and elevate chiropractic as an alternative, rather than integrated modality. So although maintaining a focus on chiropractic-specific issues was a minority opinion, it was still represented with strong language. This division in responses may parallel the schism in the chiropractic profession at large [17, 24]. Alternatively, it may be that there is some overlap between the two groups; some respondents may envision also having clinical chiropractors as well as chiropractic radiologists more integrated with mainstream healthcare so that serving chiropractors may not necessarily be antithetical to serving in mainstream healthcare. More research into the specifics on this issue, that is, the true meaning of responses and how widespread these opinions are would be useful.

### Threats

Thirty threats in four categories were identified, and each category seemed important. Five DACBRs wrote that they thought their speciality would become extinct altogether. These respondents were clear in their meaning, two used the word ‘extinct’, one wrote ‘may cease to exist’, one ‘die out’, and one ‘go the way of the dinosaur’. Three respondents related the situation to lack of embracing evidence-based care and/or lack of being accepted by the evidence-based healthcare community. One indicated the opposite, writing, ‘too many of my colleagues want to be radiologists not chiropractic radiologists.’ One gave no rationale for the prediction. It seems as if some chiropractic radiologists see a way forward, but do not think it is achievable. The most represented threat (*N* = 14) was reimbursement. DACBR services were reported as not covered by insurance companies or reimbursed at diminishing rates. This certainly represents a credible threat. If medical radiologists are reimbursed by insurance or national health plans, patients or practitioners would prefer to use them rather than pay cash to chiropractic radiologists. ‘Less radiography’ occurring in diagnostic imaging overall was also identified as a threat (*N* = 8). DACBRs have had a historical emphasis on plain radiography, which is decreasing in use. This threat can only be overcome by access to advanced imaging, with certification in interpreting these modalities. This is a challenge when most chiropractic teaching institutions, even those at universities, are not affiliated with hospitals. The final threat identified (*N* = 3) was medical prejudice. Chiropractic, as a whole, does not embrace evidence-based care in a comprehensive and well-publicised way. The evidence for this is in some of the articles cited previously in this paper, touting vitalism and subluxation-based chiropractic [3, 4, 6, 22, 28, 31]. With public statements like those, it is not surprising that some in the medical community have a negative opinion of chiropractic.

Most of the responses fit obviously as a strength, weakness, opportunity or threat, but some statements were ambiguous, and were given further consideration. Respondent 45 stated, ‘There will only be handful of chiropractic radiologists working in a full time practice solely dedicated to radiological consultation. Most will likely need a full time work whether in a clinical or academic setting.’ This was coded as a strength, under ‘teaching’ because it mentioned academics as a function of DACBRs, but it could have been listed as a weakness because it implies that the future of radiology practice for DACBRs is limited. Academic positions for DACBRs were viewed as viable for the foreseeable future. Respondent 17 wrote, ‘Some residencies will be phased out due to the cost of meeting stringent external accreditation expectations.’ This was coded under ‘opportunities’ because the theme of accreditation was secondarily coded as an opportunity. But it seems clear that the respondent viewed any new accreditation process as onerous and expected negative outcomes should that situation arise. Respondent 33 wrote, ‘Imaging groups… are less likely to hire chiropractic radiologists who can’t perform basic interventional procedures.’ This could have been coded under the primary theme of ‘integration’ or under the secondary theme of ‘threat’, but because it raised a specific opportunity for DACBRs to expand their scope of practice, it was designated its own primary code under the secondary code under ‘opportunity’. At the time of this writing, the idea of expanding the scope of practice is the subject of debate in the wider profession as well, where some have advocated for chiropractors to gain limited privileges to prescribe drugs [51–54].

Respondent 40 wrote, ‘As fewer chiropractors take x-rays, those patients who DO need x-rays will be sent to imaging centers who have their own medical radiologists.’ This was listed under ‘threats’ because it seemed to imply a lesser role for DACBRs. However, in the contexts of evidence-based practice and the fiduciary contract that healthcare professionals have with the public, it could be interpreted as a strength. This is because although chiropractors have developed evidence-based guidelines for radiography, some chiropractors have been documented as overusing diagnostic ionising radiation as well as publicly advocating for its overuse [55–64]. Only two respondents mentioned the concept of evidence-based practice, which was surprising, given its prominence in the literature and in policies. Evidence-based practice has been demonstrated to reduce hospital stay lengths, to increase survival outcomes and quality of care, and reduce the cost of care [65–70]. Its adoption by chiropractic at large would seem vital to the profession’s future.

### Limitations

The overall response rate to the survey was low at 38%, but this was within the expected realm of similar surveys [71–73]. In addition, two respondents stated that the issues were too complex to write on a survey form. In the future, focus groups or a workshop could yield more detail from participants and would allow follow-up questions for improved clarity. Some of the coding may have misinterpreted the true meaning of a respondent, and this could be improved in future studies by having more than one person code the responses on a consensus basis, similar to a systematic review. Ambiguous statements could have been double coded. This would have yielded slightly different numbers in the results, but likely would not have significantly changed outcome of the study.

## Conclusions

The historical paradigm of radiography, that is, the use of x-rays to document chiropractic subluxations, was cited in the responses to the item analysed in this paper. It seems as if very few DACBRs hold that paradigm, but a sizeable minority tolerate or defer judgement on its use by their referring chiropractors and teaching institutions. In the current era of the increasing necessity of demonstrating evidence for diagnostic and therapeutic procedures, it seems likely that chiropractic radiologists and the wider chiropractic profession will need to take a more active position promoting evidence-based practice. Re-evaluation of professional guidelines and legislation, more stringent management of continuing professional development activities, as well as improvement in accreditation policies and practices should be considered. The consequences of failing to do so include increased marginalisation and reduced viability as a profession.

## Additional file

Additional file 1: Answers to question 34. (DOCX 140 kb)
