# Supplementary material for: Historical influence on the practice of chiropractic radiology: part II - thematic analysis on the opinions of diplomates of the American Chiropractic College of Radiology about the future
Source: Chiropr Man Therap. 2017 May 8;25:15. doi: 10.1186/s12998-017-0145-z (PMC5421334; doi:10.1186/s12998-017-0145-z)
Supplement: Additional file 1: — Answers to question 34. (DOCX 140 kb) [file 12998_2017_145_MOESM1_ESM.docx]

**Additional file 1: Answers to question 34, ‘What do you think the future holds for the practice of chiropractic radiology?’**

R1: “If the profession as a whole can move towards working in an evidence-based manner, then there may be a future for chiropractic radiologists. If not, we will remain marginalised and officially unrecognised and may even cease to exist.”

R2: “I believe there is a place for chiropractic radiology especially if the referring doctor wants a more detailed report with findings sometimes overlooked by our MD radiologists brethren. Our perspective and understanding to patient management with chiropractic aids with our interpretation of imaging studies that MDs do not have.”

R3: “The discipline is going to need to evolve as digital imaging improves and becomes more prevalent. I am seeing more doctors utilize advanced imaging facilities for x-ray referrals due to equipment cost. I believe we will benefit from looking at ways to integrate into imaging facilities to access these referrals. I believe we will need to become more capable of accessing advanced imaging facility referrals for MRI, etc. from chiropractic referral sources. Teleradiology issues will need to be ironed out for our profession as private practitioners accessing digital equipment will make image transference easier and more real time. Our prompt assessments of referred digital imaging studies from private offices will assist us in our roles of providing guidance and expertise to the chiropractic profession in regards to diagnostic imaging.”

R4: “Unless chiropractic radiology can incorporate more advanced imaging into radiology practices, there will be very few chiropractic radiologists that can afford to practice as pure radiologists without also treating patients. In my geographic area, MD radiologists are pressured by their colleagues to not allow DC radiologists to practice with them. Managed care reimbursement rates for x-ray studies are too low to make a living on just x-ray without MRI or CT, etc.”

R8: “In what country? The answer differs depending on the jurisdiction.”

R10: “I believe that the chiropractic profession in general improves over time as education has improved. Albeit, this is a slow process.

Referral to chiropractic radiologists will slowly become more prevalent as chiropractors increasingly value the service and as the number of chiropractors increase. I have had many of my clients for over 10 years and their continued referrals suggest they value my service (chiropractic radiology). Point being that once a chiropractor avails themselves to my service, they almost always continue referring images if they can afford it. Lastly, specialized fields (like chiropractic radiologists) depend on the health of the referring body of chiropractors. I feel the future of the chiropractic radiologists is intimately tied to that of its profession. It takes a successful chiropractor to pay a specialist for their service.”

R11: “Some of the most notable accomplishments in recent years have come through upper cervical chiropractic. CBP authors have produced some of the most quoted articles in mainstream journals. Chiropractic radiology can support these areas and in turn support the survival of the profession. As the profession goes, so do we. As a group of imaging professionals, we do not have the numbers or the resources to directly compete with medical radiology.”

R12 “The practice will largely be dependent on financial structures, coverage and reimbursement.”

R13: “Hopefully towards the trend of being further and more widely incorporated into allopathic medical imaging centers. This needs to happen more than it does.

Additionally, one comment I have is regarding the use of [fluoroscopy] when another faculty member at the chiropractic institution wanted to bring in this consultant for the company that sells the [fluoroscopy] equipment. I whole-heartedly denied his request, as did another chiropractic radiologist working here. This is a problem as these representatives target DCs, especially new DCs, that this is the technology that is needed in their practices. And several fall victim to this which does not have any research or radiologic justification (radiation protection). I would like to see this gone - it does not offer medical benefit.”

R14: “Chiropractic radiology...unsure. As a radiologist who reads films for chiropractic practices... continuing growth.”

R15: “I'm most concerned about the level of integration we have with medical imaging centers and hospitals. As fewer private practice chiropractors install x-ray equipment in their clinics, it will be more important for us to partner with outpatient imaging centers, or even hospitals, in order for our practices to thrive.”

R16: “The future of chiropractic radiologist depends directly on the practice of chiropractic. If acceptance and respect of chiropractors as primary contact docs is maintained and DCs support and request DC radiologists to read their x-rays and advanced imaging..... Again, 20% of my full time practice is requests from health practitioners other than chiropractors. So they respect our competency and education. Chiropractors that refer to imaging centers are usually practicing at a high level of competency and expectation. There are DCs who are not Diplomates reading for imaging centers. Many of the MD radiologists are not DABRs [Diplomates of the American Board of Radiology] as well.

At one of our centers we did an anecdotal evaluation of all health fields referring to the center and positive findings on all imaging, but especially MRI. We found that DC referrals had higher percentage of positive findings justifying the study requested. General practitioners (MD) seemed to be fishing or hoping the radiologist would make the diagnosis for them, or maybe were pleasing the patient. Of course, we omitted follow-up studies by oncologist.”

R17: “Some residencies will be phased out due to the cost of meeting stringent external accreditation expectations.”

R19: “We need a strong independent DACBR professional organization to represent us a distinct speciality.”

R20: “The future is as bright and as uncertain as it is for chiropractic, in general and for most areas of healthcare...with the push towards socialized healthcare (even more radical than Obamacare), God only knows what the future is...but 25 years ago, some of the so-called experts in our group who were in the ACA leadership basically said at one meeting that we should just all chuck it in, because we'd all be cracking backs soon and there was no future at all for chiropractic radiology. I have had a rich & satisfying radiology practice over the next 25+ years and certainly hope that the younger DACBR's who work really hard will see the same kind of rewards for their hard work. Opportunities may present themselves to us, but more often than not, we make our opportunities by working our rear-end off, ethically and without compromise...and with a whole lot of patience & humility. These are the keys to success in our specialty & life, in general.”

R21: “Depends in the US on whether we get ourselves certified to read advanced imaging without overreads from MDs.”

R22: “The main challenge is the inadequate insurance reimbursement for services rendered. I think there will be a lack of growth in the profession as a result.”

R24: “Internationally, this practice is under scrutiny and continued abuse may see further restrictions placed on chiropractic "radiography", including a move towards referral to "medical" imaging centers exclusively in Medicarelike systems. Chiropractic "radiology" will survive, but will move towards 100% digital environments and probably online/distant/international interpretation services.”

R25: “This depends on the country. In Switzerland chiropractors are part of medicine with quick access to any and all imaging procedures with full reports. Therefore, chiropractic radiologists are not really needed. However, I am treated as an equal from the medical radiologists.”

R26: “It would appear to me that there are many academic leaders who are almost totally devaluing and discrediting the need for chiropractors to order or take Imaging studies. These efforts must be vigorously opposed since it is this privilege that mostly sets our system of patient care apart from all others (pists and paths). If this privilege is lost, there is precious little left for us. The ACCR must a assume a leadership role in reeducating the need for diagnostic imaging in our practices. Missing one lesion discredits the entire profession.”

R29: “It will eventually die out, not in my or your lifetime. We have too few new members each year and many of my colleagues want to be radiologists not chiropractic radiologists. Ooh you didn't expect that did you. What's the difference you say....you'll see.”

R30: “About the same, except new DACBRs should be taught to have more of a service attitude toward treating chiropractors.”

R31: “We will continue to be the red-headed stepchild of imaging. We will have to get some form of external accreditation for our training and certification process or ACR will push us out of the sandbox. They aim to own rights to all image interpretation and carriers will be glad to help them if they think will allow them to refuse claims and save them some money.”

R33: “There will always be a place for an expert consultant in diagnostic imaging for clinicians. However, I think that the profession of chiropractic radiologists will be significantly challenged in the future if it does not adapt to the market changes in diagnostic imaging. The need / demand for radiographic studies of patients with musculoskeletal conditions is progressively reduced and often directly replaced by more advanced imaging procedures. These advanced imaging equipments are much more expensive to own and prohibitive for clinicians and solo chiropractic radiologists. Doing secondary reads for clinicians or primary reads for other doctors who will sign the reports can pay the bills but is not a very rewarding enterprise. I see the future employment of chiropractic radiologists to reside primarily among imaging groups, which are less likely to hire chiropractic radiologists who can't perform basic interventional procedures. Unless chiropractic radiology residency programs adapt by increasing the training of residents in interventional procedures, and the scope of practice allows them to perform these procedures, the future employment prospect of their residents is greatly limited.”

R34: “Very poor unless you are teaching for a college. To long to explain. You may call me if you want to discuss. [phone number]”

R36: “I feel the future of chiropractic radiology is precarious at best, I would not be surprised to see the field completely disappear in 20 to 30 years. The increasing cost containment measures implemented by third party payers continually erodes the profitability of film interpretation. The relatively low number of practicing DCs that use DACBRs puts the profession at risk. Good DC practitioners are being forced out of business because of decreased profitability and they are the most likely subgroup to utilize a DACBR service. Continual efforts by organized medicine to exclude DACBRs from film reading opportunities will also make it more difficult to remain viable.”

R37: “It is a very very underrated practice and the value of which seems to be ignored by 90% of practicing Chiropractors. Most DC's believe that their 3+ years of radiology classes in undergrad and clinical experience deem them as experts in the field of radiology. The majority of questions from "one-time" clients are of an appearance that is unusual to them, but normal none the less. And certainly pathology is at times missed in entirety and without our help, would have been a missed diagnosis.”

R38: “I think (hope) there will be continued opportunities to practice radiology as a chiropractor serving providers whose method of treatment is primarily non-surgical (i.e. D.C., DPT, PMR, etc.) We have to be aware of and contribute to the current literature with respect to the significance of imaging findings so that we can accurately communicate with our referral base.”

R40: “lower reimbursement. Makes it a harder business decision to spend a substantial amount of money for less return. As fewer chiropractors take x-rays, those patients who DO need x-rays will be sent to imaging centers who have their own medical radiologists. It behooves chiropractic radiologists to affiliate with these imaging centers to overread the medical reports. I do this as part of my practice. interesting and sometimes disappointing to see what they miss.”

R41: “All professions must evolve or suffer extinction. Chiropractic radiologists didn't evolve for subluxation analysis, but as diagnostic specialists. The use of imaging technology for patient diagnosis is constantly evolving and getting more complex. Radiologists as specialists interpret these patient images and also serve as consultants for clinicians. The demand for chiropractic radiologists will grow as the chiropractic clinicians skill set grows with the management of more challenging clinical problems.”

R42: “No idea about the future, or how (at least in the US) how healthcare changes will affect it. Hope it gets better for chiro rad, ie, reimbursement gets better, but hard to imagine that. Use of mainstream radiographic guidelines in a non-mainstream profession is problematic”

R43: “This question is too broad. Respectfully, I don't have time to tell you everything I think about this topic. May I suggest you conduct focused in-person interviews to elicit answers to this question. Thank you for researching our specialty. Your work is much appreciated.”

R45: “There will only be handful of chiropractic radiologist working in a full time practice solely dedicated to radiological consultation. Most will likely need a full time work whether in a clinical or academic setting.”

R46: “Sadly, feel because insurance reimbursement is now so low that the specialty will migrate back to how it started, i.e. training radiologists to teach at the institutions.”

R47: “I don't really know what the future holds but I worry that we are not valued enough by the chiropractic profession and hence are in a vulnerable position. As a sub-specialty chiropractic radiology does not seem to be growing with the chiropractic profession as a whole and that worries me.”

R48: “Here in the USA, I have seen that fewer chiropractors are obtaining x-ray equipment for their offices when they start practice. Many long time practicing chiropractors are getting rid of their equipment. It is no longer a problem for chiropractors to refer their patients to imaging centers. My business is significantly decreased since the 1980s. The NBCE practice analysis found that only 45% of responding chiropractors had imaging equipment in their offices.”

R49: “it is going to be all digital. Less radiographs. More MRI. The chiropractic radiologist is going to have to be credentialed mainstream for reimbursement purposes. the DACBR will have to be able to add value to the imaging facility, to the care of the patient and to the referring clinician.”

R50: “New DACBR will have to come to grips with a declining reimbursement model in the US. Teleradiology and digital equipment two large areas that lack information, guidelines and state to state licensing variances. Chiropractic radiology training/certification for MRI reading lacks structure, lacks a credentialing process, etc. MSK US - same issues as MRI apply to this area.”

R51: “I think the future is bright in some countries, not in others. Completely depends on the health care system. Universal health care allows access to medical x-rays and reports for 'free', making chiro radiology a peripheral and very select choice.

Some countries prohibit chiros from taking x-rays; in these cases, chiro radiology will also likely go the way of the dinosaur. It seems most chiro offices that have x-ray suites produce garbage film quality or are doing subluxation analysis. As evidence based medicine and collaborative care continues to gain popularity, how long will it be before chiros in North America also lose their right to use (abuse) ionizing radiation as a diagnostic tool?”

R54: “"Future's so bright, I gotta wear shades". (Timbuk 3) Seriously, the outlook for chiropractic radiology as a profession is positive... we spent the decade of the 1990's achieving appropriate autonomy as a specialty, somewhat languished in the 2000's and are now (2010's) working on our legitimacy, integration and recognition as a peer group to medicine in musculoskeletal imaging through certification, expansion of credentials and accreditation as a specialty. We will have a decade or more of this to go, but I am confident we will carve out a niche in MSK imaging that is not dissimilar to our role in chiropractic educational settings.”

R55: “Reduction of use because of diminished third party re-imbursement amounts and fewer payers reimbursing chiropractors for taking radiographs.”

R56: “I think the future of chiropractic radiology can be optimistic if the teaching institutions would instill in the doctors they are training that the radiology they are learning is a basic level and they should concentrate on good film quality and let someone else (DC radiologist or other) read the x-rays they take in their office. Most pathology and more subtle fractures that I see in my office are missed by the referring doctors. They only recognize what they know... and what they know is limited. Hope this helps.”

R57: “I think it is grim. There are many radiologist now that are specializing in MSK imaging and understand biomechanics. I think we are fooling ourselves to think that the need for our specialization is going to continue.”

R58: “I think X-ray utilization is decreasing among new chiropractic graduates, but there is still a strong market for chiropractic radiology.”

R61: “The questions asked by practitioners are usually for evaluation of pathological or anomalous conditions demonstrated in images. Spinal images, if available, should be routinely evaluated for biomechanical findings as these may affect patient prognosis which is a part of the management. This evaluation should be understood within the context of positional accuracy of images and natural distortion of projectional radiography. Insufficient research has been undertaken and disseminated for professional review to demonstrate the reliability and validity of radiographic inter-segmental analysis. Ironically, orthopaedic colleagues do not appear to have similar concerns when evaluating "extended-region spinal radiographs" when evaluating for and planning scoliosis correction.”

R62: “I question the longevity of the specialty, at least in North America. The growth of medical imaging, particularly as it evolves digitally is seemingly outpacing the chiropractic profession technologically. Direct access to these technologies by chiropractic radiologists is lessened, and medicine seems happy to service chiropractors as customers. Further most chiropractors seem less inclined to pay for radiological interpretation services, and those who are inclined, many times cannot afford, the expense in a managed care model of private practice. Those practitioners who operate in a more vitalistic model are not concerned with pathology and do not believe expert interpretation worthwhile.”

R64: “Likely to become extinct.”

R65: “I feel a ethical dilemma often arises in the asymptomatic patient getting radiographic examinations on the region being treated purely for x-ray analysis. In other situations of a patient getting a regional exam for mainstream justified reasons, analysis does not represent the primary reason for the exam.”

R66: “I’m very unsure about the future of chiropractic radiology. Our existence seems very limited to academic and self-starting practitioners.”

R67: “For many years my institution priced full spine series only $10 more than two sectional studies. I successfully changed the pricing structure when I showed the clinicians were ‘super sizing’ the imaging studies by ordering TSS on patients with only cervical and lumbar complaints. I think we are remiss if we expect chiropractors to apply medical guidelines for imaging indications. We need to develop our own guidelines that take into account that chiropractors are determining the safety of introducing motion into the spine when ordering films.”

R68: “I think it is bright.”

R69: “If plain films are reduced/removed from DCs then we will see less and read less. Majority of my reads are cash basis because insurance reimbursements are so poor. Our DACBR status does not help us outside chiropractic circles and is too limited. Not being ACR certified AMA partners will continue to limit us. We are a minority in a large profession and will become extinct unless we are accepted by medical community.”

R70: “A decline in use. I don’t think the newer DCs are using marking systems as much, and many new DCs are not putting in x-ray units. I still think that contemplation of CMT can be an indication for radiographs because we cannot palpate anatomic changes but I do not see justification for follow up x-rays just to follow a series of chiropractic treatment.”

R71: “I believe we need to integrate in imaging centers as much as possible. Teleradiology has great potential for us. That said: it is imperative that ACCR gain accreditation. In my opinion chiropractic radiology can and must be the force that helps integrate the profession into medicine as a whole. Tough job, but we must set the standard and lead, because we can.”
